# Pediatric seizure-related posttraumatic stress and anxiety symptoms treated with EMDR: a case series

**DOI:** 10.3402/ejpt.v7.30123

**Published:** 2016-07-04

**Authors:** Elmedina Dautovic, Carlijn de Roos, Yanda van Rood, Agnes Dommerholt, Roos Rodenburg

**Affiliations:** 1 Research Institute of Child Development and Education, University of Amsterdam, Amsterdam, The Netherlands; 2 Kristal, Centre for Psychiatry and Intellectual Disability, Leiden, The Netherlands; 3 Psycho-trauma Center for Children and Youth, Rivierduinen, Leiden, The Netherlands; 4 Leiden University Medical Center, Leiden, The Netherlands; 5 Stichting Epilepsie Instellingen Nederland (SEIN), Heemstede, The Netherlands

**Keywords:** Children, epilepsy, anxiety, trauma, EMDR

## Abstract

**Purpose:**

To examine the potential effects of eye movement desensitization and reprocessing (EMDR) in children with epilepsy-related posttraumatic stress and/or anxiety symptoms, using a case series design.

**Methods:**

Five children (aged 8–18) with epilepsy identified for seizure-related posttraumatic stress and/or anxiety symptoms were treated with EMDR. To examine potential treatment effects, posttraumatic stress and anxiety symptoms were assessed (CRTI and SCARED) pre- and post-EMDR and at 3-month follow-up. Normative deviation scores were calculated to examine the severity of seizure-related posttraumatic stress and anxiety symptoms over time. The reliable change index was calculated for pre- to posttreatment change of seizure-related posttraumatic stress and/or anxiety symptoms.

**Results:**

Before EMDR, overall or subscale scores indicated that all children had (sub)clinical seizure-related posttraumatic stress symptoms and/or anxiety symptoms. Directly after EMDR, most children showed significant and/or clinical individual improvement, and these beneficial effects were maintained or reached at follow-up. The mean number of sessions was 2 (range 1–3, 45 min per session).

**Conclusions:**

In case of seizure-related posttraumatic stress and/or anxiety, this study indicates that EMDR is a potentially successful quick and safe psychological treatment for children with epilepsy.

**Highlights of the article:**

- The first study to examine the potential effects of EMDR to reduce clinical seizure-related posttraumatic stress symptoms and/or anxiety symptoms in children with epilepsy.
- After 1–3 EMDR (45 min) sessions, positive treatment effects were found on a range of seizure-related PTSD symptoms and/or anxiety symptoms.
- During treatment, no seizures, absences, or any other adverse events were observed; the seizure diaries showed that none of the children experienced more seizures (or an unusual pattern) after treatment.
- At the reevaluation of EMDR, all children and parents reported improvement in physical, emotional, cognitive, or social functioning.

Epilepsy is the most common central nervous system disease in children and adolescents, affecting five in every 1,000 children (Cowan, 2002). It is a heterogeneous condition, with differing etiologies resulting in recurrent seizures due to atypical electric activity in the cerebral cortex nerve cells. The condition has marked neurobiological, cognitive, behavioral, emotional, and social consequences (Mula, 2016).

Children with epilepsy are at increased risk for developing psychopathology, and these problems tend to continue into adulthood (Rodenburg, Stams, Meijer, Aldenkamp, & Deković, 2005). The prevalence of anxiety disorders in children with epilepsy is reported to range from 13.0 to 48.5%. Current prevalence rates in children from the normative population range from 8 to 21% (Beyenburg, Mitchell, Smidt, Elger, & Reuber, 2005; Jones, 2014). This may imply that some of the anxiety in children with epilepsy is seizure related (Rodenburg, Wagner, Austin, Kerr, & Dunn, 2011). For example, the unpredictable, overwhelming, and uncontrollable nature of seizures shares characteristics with traumatic events and may result in posttraumatic stress symptoms (Newsom-Davis, Goldstein, & Fitzpatrick, 1998). Seizures can also result in symptoms of social anxiety, generalized anxiety, phobia, separation anxiety, and panic. For example, children with generalized tonic–clonic seizures may develop panic due to loss of control during the seizure, or social anxiety due to having a seizure in the presence of others while incontinent for urine (Beyenburg et al., 2005).

Little is known, though, about the prevalence of specific anxiety disorders or posttraumatic stress symptoms in pediatric epilepsy (Jones, 2014). Based on a dimensional symptom inventory, 44.6% of children aged 9–14 years scored above the clinical cutoff for posttraumatic stress disorder (PTSD), while 12.5% of normal population control children scored above clinical cutoffs. However, the subsequent structured interview indicated that most children had subclinical PTSD, that is, not meeting all the criteria for PTSD (Dunn, Austin, & Perkins, 2009). Kotwas et al. (2016) reported PTSD in 2% of the children with epilepsy. For panic, 36.5% of children aged 9–14 years appeared at risk, in contrast to 9.9% of normal control children (Dunn et al., 2009).

Anxiety and stress reactions not only increase the burden of epilepsy but can also trigger and increase the frequency of seizures (Lathers & Schraeder, 2006). That is, anxiety is perceived by people with epilepsy as a precipitating factor of seizures and impacts via neurophysiological and hormonal changes, which are related to stress hormones, on neuronal excitability and as a consequence, the susceptibility for seizures is increased (Kotwas et al., 2016). Although effective treatment is required, specific evidence for treatment effects for children with epilepsy who have seizure-related posttraumatic stress or anxiety symptoms is still lacking (Jones, 2014).

As yet, treatment guidelines describe trauma-focused cognitive behavioral therapy as treatment of choice for PTSD. An intervention aimed at processing unresolved memories of negative experiences is eye movement desensitization and reprocessing (EMDR), a promising (AACAP, 2010; NICE, 2005) but yet not recommended treatment. Meta-analytic results showed that EMDR is an effective treatment for PTSD in adults and children (Chen et al., 2014; Rodenburg, Benjamin, de Roos, Meijer, & Stams, 2009).

EMDR allows to work in a non-verbal manner. This is probably advantageous for children with epilepsy in whom the somatic components of the targeted memories play an important role. Until now, only two case studies have reported the use of EMDR in (young) adults with epilepsy and comorbid psychopathology. One case study of a 34-year-old sexually abused woman with PTSD and comorbid epilepsy showed that after six EMDR sessions intrusions, anxiety and depressive mood improved significantly (Schneider et al., 2005). During the night following the second session, she had a reactivation of idiopathic generalized epilepsy with multiple seizures. However, it seemed likely that this had been induced by lack of sleep, emotional distress, and benzodiazepine withdrawal. An 18-year-old boy with epilepsy and mild intellectual disability treated with EMDR for posttraumatic stress symptoms related to domestic violence was successfully treated in five sessions. One seizure was reported during admission before the start of EMDR and was probably related to non-adherence to the medical treatment while at home. No further adverse effects were reported (Rodenburg, Benjamin, Meijer, & Jongeneel, 2009).

The present case series aims to examine whether EMDR directed at processing seizure-related disturbing memories reduces posttraumatic stress symptoms and anxiety in children and adolescents with epilepsy. It is hypothesized that EMDR treatment will result in a reduction of seizure-related posttraumatic stress and anxiety symptoms from (sub)clinical to non-clinical levels. This study also explores whether EMDR treatment can be safely delivered to children with epilepsy. Safe delivery is defined as no marked increase of seizures during and following EMDR sessions, that is, not an increase in seizures from the earlier general pattern of seizures nor an increase of severity of seizures (Michaelis et al., 2016).

Although EMDR is an effective treatment for PTSD, the clinical significance and utility of EMDR as delivered in pediatric epilepsy practice has not yet been demonstrated. To study the potential effects of EMDR treatment in this special clinical population, we opted for a case series design (Drotar, 2009; Drotar, La Greca, Lemanek, & Kazak, 1995). Case series allows to investigate the course of symptoms over time in relation to the broader clinical overview (e.g. Hurtado-Parrado & López-López, 2015) and when repeated, contributes to clinical generality as the same phenomenon is repeatedly investigated in new patients (Branch & Pennypacker, 2013). This is a repeated series of cases conducted in routine pediatric epilepsy clinical practice using the standard EMDR protocol for children. As such, the data are practice-generated, which allow clinicians working in the field to easily apply the used procedures to their own patients (Hurtado-Parrado & López-López, 2015).

## Methods

### Procedure and participants

This study was part of a larger project of the Epilepsy Institute in the Netherlands Foundation (SEIN) and the University of Amsterdam (UvA) on seizure-related posttraumatic stress and anxiety symptoms in children with epilepsy. The project was approved by the Medical Ethical Committee of the Academic Medical Center of the UvA (MEC 10/008 # 10.17.0033as). The larger project consisted of (1) a screening study on seizure-related posttraumatic stress symptoms and/or anxiety and (2) a treatment study in which children with clinically elevated levels of PTSD and/or anxiety and for whom a relationship with epilepsy and/or seizures was established were treated with EMDR. Participants were children with epilepsy and their parents, seen at the outpatient clinic of epilepsy center Stichting Epilepsie Instellingen Nederland (SEIN) for routine consultation between March and June 2010, and all provided written informed consent. Prior to the routine consultation, children and parents received an information letter about the study at home. Before routine consultation with the treating child neurologist, participating children and parents completed a questionnaire package in a separate room. Children were asked to complete questionnaires on posttraumatic stress symptoms, anxiety symptoms, and their epilepsy at pretest one week before treatment (T1), at posttest one week after closure of EMDR treatment (T2), and at 3-month follow-up after treatment (T3). The data were collected by the first author of the study, not by the treating EMDR therapists as to separate study results from the treatment itself. Parents helped keeping a seizure diary.

Children were included if they were aged 8–18 years, were diagnosed with epilepsy, had an estimated IQ≥75, were at that time treated for epilepsy by a child neurologist, and had at least subclinical (1 SD above mean) levels of anxiety (on at least 1 anxiety subscale), and/or posttraumatic stress symptoms. Excluded were children with chronic conditions (physical and psychological) other than epilepsy (with the exception of ADHD which is common in children with epilepsy; Dunn et al., 2009), children currently with a history of psychogenic pseudo-epilepsy seizures, and children without (≤1 SD above the mean) posttraumatic stress symptoms and/or anxiety.

Children with pretreatment scores indicating at least subclinical levels of posttraumatic stress and/or anxiety symptoms were referred for an intake session with a child psychologist.

With these symptoms as a point of reference, using the DSM-IV criteria for PTSD or anxiety disorder, the child psychologist examined whether etiological and subsequent aggravating seizure-related events could be meaningfully identified on a time line and whether a relationship between symptoms and these experiences could be established (De Jongh, Ten Broeke, & Meijer, 2010). The following question was asked to the child and parent to establish this relationship: “which event or events may have caused the current complaints or might have worsened them.” EMDR treatment was directed toward those identified and meaningful memories related to the existing complaints, which were also emotionally disturbing in the present. When the child's symptoms were within the (sub)clinical range, but not related to the seizures, children and parents were referred to appropriate mental health care.

Children diagnosed with seizure-related posttraumatic stress or anxiety symptoms were eligible for the EMDR treatment study and were (if they consented) referred to one of two experienced EMDR therapists. The Dutch standard EMDR protocol for children and adolescents until 18 years was used for this study (Beer & de Roos, 2004).

### Treatment: EMDR

The EMDR procedure consists of eight stages. After taking the history, explaining EMDR, and identifying the most distressing seizure-related memories (i.e., target selection), the target image(s) are processed consecutively. The therapist asks the patient to hold the disturbing target memory and aspects related to it in mind and to simultaneously attend to a distractive task introduced by the therapist. The child is asked to follow the therapist's finger making saccadic movements at a rate of about two-stimuli/s for about 30 s. In the case of problems with eye movements, auditory bilateral stimulation or tactile stimulation was used. The child is then asked to briefly report what comes to mind (associations). The procedure is repeated until the original target is no longer disturbing and dysfunctional cognitions about the trauma have become functional (Shapiro & Laliotis, 2015).

In this study, one EMDR session lasted 45 min and the preset maximum number of sessions was five. However, the actual number of sessions could vary according to the amount needed to desensitize the distressing target memories, as indicated by the child's self-reported distress score of 0 for each memory, as scored on a ten-point Likert-scale (Subjective Units of Disturbance). In light of safe delivery of EMDR, if increases in seizures during the sessions would happen, adaptations to the EMDR procedure could be made by shortening the session duration or by changing the type of external stimuli (auditory or tactile stimulation).

A total of 82 children and their parents were invited to participate and, of these, 38 agreed (46.3%). Of the non-participating children, 34 refused participation (41.5%) and 10 (12.2%) were excluded because of inconclusive EEGs and uncertainty considering their epilepsy diagnosis (*n=*4), comorbidity with mild/moderate intellectual disability (*n*=4), autism spectrum disorders (*n*=1), and insufficient understanding of the Dutch language (*n*=1). Of the participating families, seven failed to return their questionnaires. In total, 31 children participated: nine boys (29.0%) and 22 girls (71.0%). Of these, 12 (38.7%) children had (sub)clinical elevated posttraumatic stress symptoms and/or anxiety. Of these 12 children, four refused an intake session because they thought they did not need treatment. Seven children scored above (sub)clinical cutoffs: posttraumatic stress symptoms (*n*=1), anxiety symptoms (*n*=1), or both (*n*=5).

Finally, six children and their parents agreed to participate in the present EMDR study. For five children, during an intake session prior to EMDR, the child psychologist confirmed a relationship between posttraumatic stress and/or anxiety symptoms and the seizures (Table 1). For one child, there was no such relationship present and she was referred to general mental health care.

**Table 1:** Overview of the five participants in the EMDR treatment study

Demographic and diagnostic information							

Child ID no.	Sex	Age (years)	Ethnic background	Education	Seizure type	Seizure frequency	Relationship between seizures and PTSD/anxiety^a^
1	M	9	Dutch	Special needs elementary school	Tonic–clonic seizures	Seizure-free^b^	3
2	F	12	Dutch	Special needs elementary school	Absences	Daily	3
3	F	12	Dutch	Regular elementary school	Tonic–clonic seizures	Monthly	1
4	F	16	Turkish	Vocational education	Complex partial seizures	Weekly	2
5	F	15	Dutch	Vocational education	Tonic–clonic seizures	Seizure-free^b^	3

Note:

a 1=seizure-related anxiety; 2=seizure-related trauma; 3=both seizure-related.

b Seizure-free means without current seizure activity, a child may however follow an anti-epileptic drug regime. Seizure-free does not mean that the diagnosis of epilepsy is not valid anymore. According of the new definition of the International League Against Epilepsy (ILAE), a diagnosis of epilepsy would be reconsidered if the patient is in remission for the last 10 years, from which the recent last 5 years off all anti-epileptic drugs (Fisher et al., 2014).

### Measures

#### Posttraumatic stress symptoms

The Children's Responses to Trauma Inventory (CRTI) (Alisic, Eland, & Kleber, 2006), based on the Impact of Event Scale (IES) (Horowitz, Wilner, & Alvarez, 1979) and the Impact of Event Scale Revised (IES-R) (Weiss & Marmar, 1997), was used to assess child posttraumatic stress symptoms. This questionnaire consists of the subscales intrusion, avoidance and arousal, and also addresses other specific trauma reactions.

In accordance with Alisic et al., the introductory question of the CRTI was slightly adapted to examine whether children displayed seizure-related posttraumatic stress symptoms. The index trauma at the start of the questionnaire was predetermined: “Have you ever experienced anything shocking/upsetting/scary related to your epilepsy and/or seizures? If not, you do not have to complete this questionnaire.” Children were asked to remember the seizure-related traumatic event(s) and to rate on a five-point Likert scale (0=not at all; 5=often) how often symptoms occurred during the previous week. The reported reliability (Cronbach's α) is 0.92; the subscale reliability ranges from 0.71 to 0.79 (Alisic et al., 2006).

#### Anxiety symptoms

Child-reported symptoms of the entire anxiety spectrum were assessed with the Screen for Child Anxiety Related Emotional disorders (SCARED-R) (Muris, Mayer, Bartelds, Tierney, & Bogie, 2001). The SCARED-R is sensitive to treatment effects (Muris et al., 2001) and consists of 69 items to measure generalized anxiety disorder, separation anxiety including school phobia, social phobia, panic disorder, obsessive–compulsive disorder, PTSD, and three specific phobias: the animal type, the blood–injection–injury type, and the situational or environmental type. Children were asked to rate on a three-point Likert scale how often anxiety occurred (0=almost never; 1=sometimes; 2=often) with regard to the previous week. The reported reliability (Cronbach's alpha) of this scale is 0.92 and the subscale reliability ranges from 0.66 to 0.87 (Muris et al., 2001).

#### Seizure diary

As seizure occurrence was deemed important in light of safe delivery of EMDR, seizure diaries to keep track of seizure occurrence (yes/no) were kept by the parents and the children during the treatment and follow-up period. Parents were instructed to report seizures or side effects between sessions, at posttreatment, and at follow-up, and therapists were instructed to note any possible side effect during sessions.

### Statistical analysis

Normative deviation scores (NDSs) were calculated to obtain the clinical status of the child, that is, the severity of their problems before treatment (Veerman, 2007). The NDS indicates how much an individual score deviates from the normative score (norms of the respective measure used). An NDS ≤1.00 indicates no problem, ≥1.29 indicates considerable problems, and ≥1.96 indicates very serious problems compared to the normative population.

The reliable change index (RCI) was calculated. The RCI is a statistical test to examine whether pre- to posttest changes for posttraumatic stress symptoms and/or anxiety are significant or not per respective child (Jacobson & Truax, 1991; Veerman, 2007). The RCI controls for coincidence or error. An RCI of 0 indicates that no difference is detected between pretreatment and posttreatment scores, whereas an RCI of 1 indicates that the difference between the pretreatment and posttreatment scores is equal to the standard error of its difference. The RCI is considered to have a normal distribution with a mean of 0 and an SD of 1. Based on α=0.05 or based on α=0.025 (one-tailed significance testing), RCIs of >+1.64 or <−1.64, and >+1.96 or <−1.96, respectively, indicate a significant change. A clinically significant change indicates a decrease from clinical to non-clinical levels. A combination of both a clinical and a significant change suggests both a real and a relevant change (Jacobson & Truax, 1991).

## Results

### Reduction of seizure-related posttraumatic stress symptoms and anxiety from (sub) clinical to non-clinical levels

#### Overall changes

Table 2 shows the changes in overall PTSD symptoms for the five children. Prior to EMDR, two children had subclinical and another child had clinical overall PTSD; these scores were significantly diminished after EMDR and remained non-clinical at follow-up.

**Table 2:** Normative deviation score and reliable change index of PTSD symptoms (from the CRTI)

Overall PTSD symptoms (CRTI)					

Child	NDS pretest	NDS posttest	NDS follow-up	RCI pretest-posttest	RCI posttest-follow-up
1	0.97	−1.12	−1.70	3.67**	1.03
2	1.08^a^	−0.93	−0.46	3.55**	−0.83
3	0.52	0.14	−0.89	0.68	1.81*
4	1.08^a^	−0.50	−0.76	2.79**	0.45
5	1.63^b^	−1.06	−1.14	4.76**	0.15

Note:

* α=0.050

** α=0.025; a=moderate problems (NDS 1.00–1.28); b=considerable problems (NDS 1.29–1.64); (NDS>1.96); RCI +1.64/+1.96 indicates positive change; RCI−1.64 to +1.64/−1.96 to +1.96 indicates no change; RCI<−1.64/−1.96 indicates negative change.

NDS: normative deviation score; RCI: reliable change index; PTSD: posttraumatic stress disorder; CRTI: Children's Responses to Trauma Inventory.

Total anxiety was subclinical for Child 1 before EMDR but showed no significant improvement post-EMDR or at follow-up. Although Child 3 had non-clinical anxiety prior to EMDR, clinical anxiety was registered post-EMDR; however, this level was significantly reduced at follow-up (Table 3).

**Table 3:** Normative deviation score and reliable change index of anxiety (from the SCARED-R)

Overall anxiety					

Child	NDS pretest	NDS posttest	NDS follow-up	RCI pretest-posttest	RCI posttest-follow-up
1	0.82	−0.26	0.14	1.24	−0.45
2	0.11	−1.28	−0.78	1.59	−0.57
3	−0.50	1.10^a^	−1.39	−1.83*	2.86**
4	0.05	−1.89	−1.89	2.22**	0
5	0.10	−0.81	−0.27	1.05	−0.62

Note

* α=0.050

** α=0.025; a=moderate problems (NDS 1.00–1.28); RCI +1.64/+1.96 indicates positive change; RCI −1.64 to +1.64 /−1.96 to +1.96 indicates no change; RCI <−1.64 /−1.96 indicates negative change.

NDS: normative deviation score; RCI: reliable change index; SCARED-R: Screen for Child Anxiety Related Emotional Disorders.

Because the (sub)clinical symptoms on the subscales of the CRTI and SCARED-R differed between the children, changes in symptoms from pre- to post- and to follow-up of EMDR are presented separately for each child.

#### Specific symptomatology: individual trajectories of seizure-related PTSD and anxiety

**Fig. 1:**
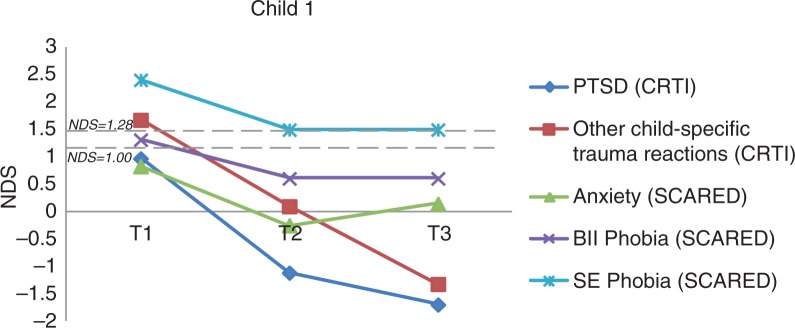
The course of (sub)clinical PTSD and anxiety symptoms for Child 1 (overall and subscale scores). *Note*. NDS: normative deviation score; BII phobia: blood–injection–injury phobia; SE phobia: situational–environmental phobia.

***Child 1*.** Child 1 was a 9-year-old boy diagnosed with tonic seizures, who was seizure-free at the time of the study. He had his last seizure 1 year and 11 months before receiving EMDR. At the time of the study, he was on withdrawal of lamotrigine. This boy had seizure-related anxiety/posttraumatic stress symptoms and remembered as most upsetting event having a seizure at age 4 when playing in the sandbox at school. During the seizure (which was accompanied by alarm, panic, and subsequent stigma), his friends covered him with sand: “I could not move, I felt as stiff as a shelf and very scared.” Also, he fell down the stairs and onto the street while playing soccer when he had a seizure. Prior to EMDR, he reported clinically significant trauma reactions (CRTI), clinically significant phobia, that is, blood–injection–injury type and situational–environmental type (SCARED). Other specific complaints were fear for falling, tonic muscles to prevent falling, wobbly legs, and inability to cycle alone and climb stairs alone. Furthermore, he felt dependent and uncertain: requested help with brushing his teeth, wiping his buttocks, and eating. Also, he was bullied by his peers and showed a delay in social, emotional, and motor development. After two sessions of EMDR, this child showed a clinical and significant decrease in seizure-related child-specific trauma reactions (RCI=3.27; *p*<0.025). Furthermore, he had a clinical reduction in symptoms of blood–injection–injury phobia (Fig. 1), which was maintained at follow-up; however, this change was not significant. Also, despite a decrease in the situational–environmental phobia symptoms, this was not a clinical or significant improvement. At follow-up, he was more independent and assertive and had more self-confidence. The bullying stopped, he was able to climb the stairs alone and even played soccer with his peers.

**Fig. 2:**
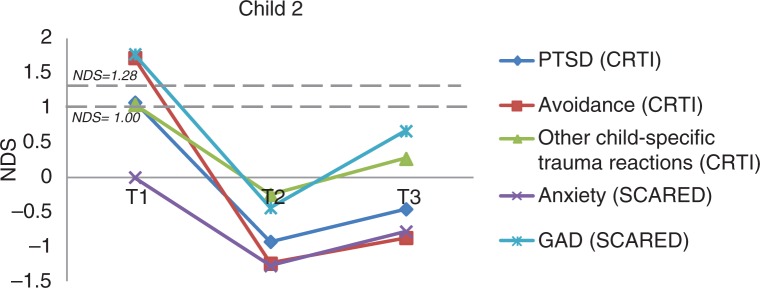
The course of (sub)clinical PTSD and anxiety symptoms for Child 2 (overall and subscale scores). *Note*. NDS: normative deviation score; GAD: generalized anxiety disorder.

***Child 2*.** Child 2 was a 12-year-old girl diagnosed with absence seizures, which occurred daily at the time of the study and for which she was partly pharmaco-resistant. At the time of the study, she was on an antiepileptic drug regimen of ethymal and valproic acid. She remembered as most upsetting event cycling and having a collision with a woman on a bike (about 3.5 years ago) who started yelling and scolding her. This still upsets her: “What if this happens with a car?” Prior to EMDR, she reported overall subclinical PTSD, clinical avoidance symptoms, subclinical-specific trauma reactions (CRTI), clinical generalized anxiety disorder, and subclinical-specific phobia: animal type (SCARED-R). Specific complaints prior to EMDR included anxiety related to losing her friends because of the epilepsy and of being bullied again. She was afraid of being perceived as “weird” by others because during her (complex partial) seizures she can act strangely, for example, excessive spluttering. She also experienced having wet trousers after a seizure in front of other children and was locked in the school toilet. After two sessions of EMDR, this girl showed a clinical and significant reduction in avoidance symptoms (RCI=3.09; *p*<0.025), which was maintained at follow-up, and a clinical and significant reduction in seizure-related general anxiety symptoms (RCI=1.96; *p*<0.05), which was also maintained at follow-up (Fig. 2). After EMDR, she could talk easier with others and had less difficulty in making contact. She also experienced more fun and reported that the tension and anxiety had disappeared.

**Fig. 3:**
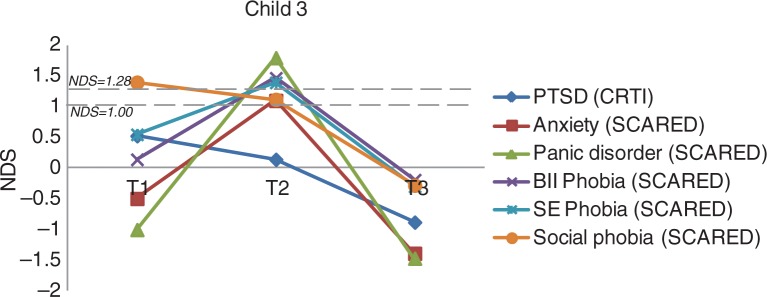
The course of (sub)clinical PTSD and anxiety symptoms for Child 3 (overall and subscale scores). *Note*. NDS: normative deviation score; BII phobia: blood–injection–injury phobia; SE phobia: situational–environmental phobia.

***Child 3.*** Child 3 was a 12-year-old girl diagnosed with tonic–clonic seizures occurring regularly since she was 2 years old. At the time, she was on antiepileptic drug regimen. The most upsetting events were (1) having a seizure while riding her bike and the subsequent fall flat on her face, causing considerable pain; (2) the fall off the horse while horse riding; and (3) a seizure after having iodine applied on a cut. Her fear of seizures started after the fall from her bike; the anxiety seemed to have worsened due to other experiences related to having seizures. Prior to EMDR, she reported clinical social phobia (SCARED-R), which was significantly decreased at follow-up (RCI=1.65, *p*<0.05). Post-EMDR, she showed an unexpected clinical and significant *increase* in panic disorder (RCI=−2.69, *p <*0.025), overall PTSD (RCI=0.68, *ns*), specific phobias of both the blood–injection–injury type (RCI=−1.38, *n.s*) and the environmental type (RCI=−0.90, *n.s*) (Fig. 3). However, these increased anxiety symptoms had disappeared at follow-up (panic disorder: RCI=3.09, *p<*0.025; overall PTSD: RCI=1.81, *p*<0.05; specific phobia of the blood–injection–injury type: RCI=1.73, *p<0*.05; and environmental type: RCI=1.81, *p<*0.05). After two sessions of EMDR, she reported that she had learned to “be herself” while horse riding. Also, she reported to feel happy and relieved and more often thinks: “I can do this.” She even dared to raise her finger in class in response to the teacher's request to join in with reading out loud. Things were going much better: she no longer feared falling off the horse and was more relaxed. Also, she less often thought about scary things and about getting another seizure. She was more decisive and had more self-confidence.

**Fig. 4:**
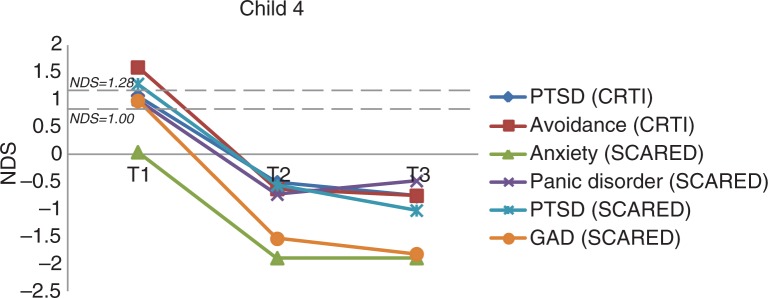
The course of (sub)clinical PTSD and anxiety symptoms for Child 4 (overall and subscale scores). *Note*. NDS: normative deviation score; GAD: generalized anxiety disorder.

***Child 4*.** Child 4 was aged 16 years, with complex partial seizures since 3.5 years which occurred weekly. She was on an antiepileptic drug regimen of keppra and lamotrigine. During a seizure, she continues to talk in an incomprehensible manner. She often scolded others during the seizures which (later) makes her feel ashamed. Regarding an upsetting event, she reported that she was not aware of such an event: “If I experience something I don't know it exactly, I only know about it from my family and friends.” She was bullied because she has epilepsy, and felt shame and withdrawal due to the epilepsy. She was anxious about going outside alone (if a seizure occurs, others will see her), was socially isolated, and was worried about scolding people during a seizure and that people might be angry or hit her. Prior to EMDR, the CRTI indicated clinical avoidance and the SCARED-R showed subclinical panic symptoms and clinical PTSD (Fig. 4). After three sessions of EMDR, avoidance symptoms (RCI=3.22, *p<0*.025) were clinically and significantly reduced, and there was a clinical and significant reduction in the panic symptoms (RCI=1.64, *p<*0.05) and in the generalized anxiety symptoms (RCI=2.28, *p<*0.025). These positive results were maintained at follow-up. After three sessions of EMDR, she could concentrate more and was happier. She did not think about the bullying anymore and, although she did not want to meet the bullies, she did go outside with her friends.

***Child 5*.** Child 5 was a 15-year-old girl diagnosed with tonic–clonic seizures, who was seizure-free at the time of the study. She had her last seizure 4 years prior to the study, but was on active antiepileptic medication at the time of the study (lamotrigine). She remembered as most upsetting event having a seizure in/near the swimming pool during her vacation: “I thought I was going to die” and being taken to the hospital by an ambulance, where everybody panicked and she could not understand the nurses and doctors. Prior to EMDR, she had clinical levels of avoidance symptoms (CRTI), and she had clinical panic symptoms (SCARED-R). Specific other complaints at intake were bad appetite, dizziness, nausea, and tiredness.

**Fig. 5:**
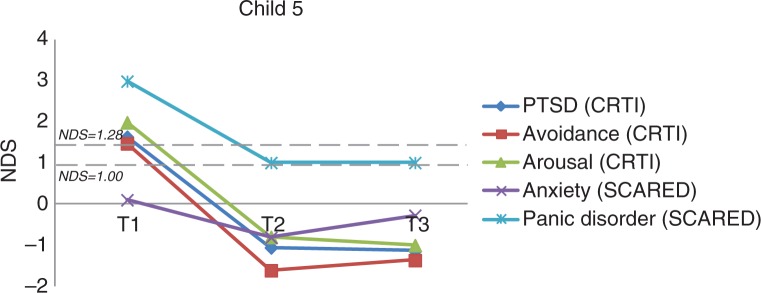
The course of (sub)clinical PTSD and anxiety symptoms for Child 5 (overall and subscale scores). *Note*. NDS: normative deviation score.

After 1 session of EMDR, there was a clinical and significant reduction in the avoidance (RCI=3.22, *p<*0.025) and arousal symptoms (RCI=2.59, *p<0*.025); this was maintained at follow-up (Fig. 5). Also, the severe seizure-related panic symptoms (RCI=1.87, *p<0*.05) were clinically and significantly reduced. She felt more energetic and the anxiety for seizures had disappeared; she felt more relaxed and had a normalized sleeping pattern, good appetite, and improved mood and concentration. Furthermore, dizziness and nausea had disappeared.

In Table 4, the EMDR process per child and per target memory is displayed.

**Table 4:** EMDR process per child and per target memory

Child	Session and target number	Target memory	Negative cognition	Positive cognition	Emotion	Body location	Sequence and intensity of subjective units of disturbance	Validity of cognition (assessment and installation phase)	Seizures before, during, or after session	Side effects
Child 1, boy, 9 years	1.1	Seizure in the sandbox at age 4, covered with sand by peers and not able to move, stiff	I cannot handle it	I can handle it	Fear	Left side of the body, shoulder, arm, back, hip and head	10-4-2-0	4-7	None	Headache during session
	2.1	Tonic seizure on the stairs and subsequent fall	I cannot handle it	I can handle it	Fear	Head	4-2-0	5-7	None	Headache and pressure in head during session
Child 2, girl, 12 years	1.1	After a seizure, at the playground, in middle with wet trousers, children laughing and yelling at her	I am worthless	I am ok	Sadness and fear	Head and heart	10-5-0	1-7	None	
	1.2	Seizure while hop-scotching, children yelling, laughing and pointing at her	I am worthless	I am ok	Sadness	Heart and belly	10-0	1-7	None	
	1.3	Locked up in the toilet by others, not daring to go out, afraid nobody missed her or asked for her	I am stupid	I am ok	Angry and sadness	Head	10-0	1-7	None	Drowsiness after the session
	2.1	Seizure while cycling, collides with a woman who yells at her	I am in danger	I am safe now	Sadness	Belly	10-0	1-7	Absence before the start of the session	
	2.2	Seizure while cycling, brother collides with her bike, father yells	I am guilty	I did what I could	Sadness	Belly	10-5-0	5-7		Tired in the days after the session
Child 3, girl, 12 years	1.1	Seizure while cycling and subsequent fall	It is my fault	I did what I could	Sadness	Head	3- 4-3-2- 1-0	4-5-7	None	
	1.2	Seizure while horseback-riding, feeling ill and having to stop riding	I am powerless	I can handle it	Sadness	Head	3-0		None	Very tired after the session
	2.1	Seizure induced after applying iodine on a cut, seeing a man from the emergency room	I am helpless	I can handle it	Angry	Head	4-0-0	5-7	None	A little tired after the session
Child 4, girl, 16 years	1.1	Seizure related bullying at elementary school, peer saying that she is having a ghost inside	I am powerless	I can handle it	Sadness	Brain	7-6-6-6-9-5-5-7-3	4	None	None
	2.1	Same target as session 1	I am powerless	I can handle it	Sadness	Brain	3-3.5-1-0	7	None	None
	3.1	Seizure related bullying at high school, seeing the head of the boy who repeatedly calls her ghost	I am powerless	I can handle it	Sadness	Head	2-1-0	1-7	None	None
Child 5, girl, 15 years	1.1	Seizure at the swimming pool while alone and not able to call for help, thought she was dying	I cannot handle it	I can handle it	Fear	Arms and legs	6-3-2-1-0	4-6-7	None	None

### Can EMDR be safely delivered to children with epilepsy?

During and after treatment, none of the children showed a decrease in seizure frequency and severity. However, none experienced more (severe) seizures than usual, as reported by parents in the seizure diaries (Table 1). Three children reported side effects of EMDR including headache, tiredness, and drowsiness.

## Discussion

This is the first study to examine the potential effects of EMDR in reducing clinical seizure-related posttraumatic stress symptoms and/or anxiety symptoms in children with epilepsy. To our knowledge, no studies have examined the use of EMDR in the treatment of children with epilepsy or for the treatment of seizure-related PTSD and/or anxiety symptoms. Our hypothesis was that EMDR treatment would result in a reduction of seizure-related posttraumatic stress and/or anxiety symptoms from (sub)clinical to non-clinical levels.

In line with our expectations, after 1–3 EMDR sessions, positive treatment effects were found on a range of seizure-related PTSD symptoms and/or anxiety symptoms. More specifically, three children with seizure-related (sub)clinical PTSD symptoms showed a significant decrease in overall PTSD symptoms after EMDR. For all children, one or more specific (sub)clinical PTSD and/or anxiety symptom(s) was significantly reduced to non-clinical levels. One girl showed an increase in specific anxiety symptoms (symptoms of panic, blood, injection, and injury phobia and situational and environmental phobia) after EMDR, which decreased to non-clinical levels at follow-up; this increase in symptoms may have been provoked by stress due to hospital admission of her mother during that period.

During treatment, no seizures, absences, or any other adverse events were observed. Furthermore, the seizure diaries showed that none of the children experienced more seizures (or an unusual pattern) after treatment. It thus seems that, in concurrence with the studies of Rodenburg, Benjamin, Meijer et al. (2009) and Schneider et al. (2005), EMDR can be safely delivered to children with epilepsy. Side effects were reported for three children: one parent reported that their child was more fatigued after treatment (a common side effect of EMDR), one child reported a headache during EMDR, and one child reported drowsiness after the session. No other side effects of EMDR were reported.

Noteworthy of the EMDR assessment phase was that seizures often had resulted in dysfunctional cognitions in the domain of control (*n=*4) and self-worth (*n=*2), for example, “I am powerless” or “I am stupid.” These domains were assessed during the EMDR procedure in terms of dysfunctional cognitions related to the target memory. One child felt still in danger after reactivation of the memory (domain of safety). The emotional response varied. Anxiety, sadness, and anger all were mentioned. The head was the place in the body where tension was felt most often (*n=*4), which seems fitting for seizure-related traumas. In most children, the Subjective Units of Disturbance of a specific memory was reduced quickly to zero within one session. One girl (girl 4) needed a second session to work on the same memory. However, three memories were treated successfully in one session in girl 3. The mean number of (45 min) sessions that was needed to process the seizure related events was 2. In that time and without parental coaching, a variety of PTSD and anxiety symptoms were reduced and the results were maintained at follow-up. Besides these results, all children and parents reported at the reevaluation of the treatment improvement in physical, emotional, cognitive, or social functioning.

An important limitation is that assessment of PTSD and anxiety was not based on a structured clinical interview (such as the Anxiety Disorders Interview Schedule-Child version (ADIS-C); (Siebelink & Treffers, 2001; Silverman & Albano, 1996). This would have provided more conclusiveness about the existence of the clinical disorders. However, the purpose of the current study was to examine whether EMDR would lead to overtime change in seizure-related posttraumatic stress symptoms and/or anxiety. Another limitation of this study is that the anxiety and PTSD symptom scales used (albeit with good psychometric properties) were not specifically developed to measure seizure-related posttraumatic stress symptoms and/or anxiety, that is, these instruments may not fully capture all aspects of seizure-related PTSD and anxiety symptoms (Holmbeck et al., 2008). During intake and treatment, some specific complaints emerged that were not covered by the questionnaires used to assess PTSD and anxiety symptoms. This implies that new and specific measures need to be developed for seizure-related PTSD and anxiety symptoms to cover more detailed treatment effects. Although this study used pre-, post-, and follow-up measurements with reliable and valid measures, used an independent assessor and manualized treatment, only five patients were included; moreover, we were unable to include a control group that received another well-established treatment for anxiety disorders to treat seizure-related anxiety and PTSD (Chorpita et al., 2002). Cognitive behavioral therapy is usually mentioned as effective therapy for treating anxiety in people with epilepsy (e.g. Jones, 2014), and given the results of this study, it would be worthwhile to study the effects of EMDR and cognitive behavioral therapy in the treatment of seizure-related anxiety. Randomized controlled studies with sufficiently large groups of patients should be conducted to further examine treatment effects in comparison with relevant control conditions and with control for time effects and other possible confounders. In addition to randomized controlled trials that are considered the gold standard for determining treatment efficacy, larger case series can also be a valuable addition to investigate symptom course over time during treatment under standardized conditions (Borckardt et al., 2008; Chorpita et al., 2002). This study may tentatively add evidence for EMDR as a treatment for seizure-related PTSD and anxiety symptoms (Chorpita et al., 2002).

Future studies should investigate seizure-related anxiety and/or posttraumatic stress symptoms in a larger and more diverse sample (e.g., more boys, more children with uncontrollable/active epilepsy of with higher seizure frequency), and by using adapted multimodal measurements. Also, daily PTSD and anxiety symptoms could be examined with regard to their role as emotional triggers of seizures and the frequency of seizures (Haut, Hall, Masur, & Lipton, 2007; Lanteaume et al., 2012). This may reveal not only treatment effects on daily PTSD and anxiety symptoms, but also whether seizures occur less frequently (Kotwas et al., 2016; Van Eerde, 2014). In this light, it would also be worthwhile to examine whether differential treatment effects occur as a function of disease activity, such as in children with refractory epilepsy or in children with reflex epilepsy (i.e., seizures induced by certain stimuli, e.g., visual or auditive stimuli). It is imaginable that more severely impaired health status is possibly associated with more severe PTSD and anxiety. Although we deem treatment effects in such populations to be similar to the effects found in our study, it may also be that more distressing memories are present and therefore the need for more sessions. Future research would benefit from the inclusion of such severe clinical populations to examine the effects of EMDR. Furthermore, a possible explanatory factor for more severe symptoms is earlier pathology in the child and parent, such as the presence of parental PTSD, which may exacerbate PTSD symptoms in the child or hamper recovery from treatment (Nugent, Ostrowski, Christopher, & Delahanty, 2007). Therefore, the interactions between treatment effects of parental PTSD and child PTSD should be studied as well. Regarding practical implications, clinicians should be aware of the existence of seizure-related posttraumatic stress symptoms and/or anxiety. Early signaling of seizure-related distress in children and referral for treatment might prevent problems continuing into adulthood, as children without PTSD and/or anxiety are less vulnerable for new upsetting events. Clinicians could motivate children and their parents to opt for EMDR treatment because it, tentatively, appears to be a safe, brief, and beneficial form of treatment.

In conclusion, in our group of children with seizure-related PTSD and anxiety symptoms, EMDR effectively, safely, and quickly reduced these symptoms to (often) non-clinical levels. Child and parent feedback of physical, cognitive, emotional, and social functioning showed fast improvement. This improvement warrants further attention from those involved in pediatric epilepsy. Further studies into seizure-related anxiety and PTSD are encouraged as to ultimately improve the care for children with epilepsy, thereby contributing to ameliorated quality of life.
